# The health literate health care organization 10 item questionnaire (HLHO-10): development and validation

**DOI:** 10.1186/s12913-015-0707-5

**Published:** 2015-02-01

**Authors:** Christoph Kowalski, Shoou-Yih D Lee, Anna Schmidt, Simone Wesselmann, Markus A Wirtz, Holger Pfaff, Nicole Ernstmann

**Affiliations:** Institute for Medical Sociology, Health Services Research and Rehabilitation Science, Faculty of Human Science and Faculty of Medicine, University of Cologne, Eupener Strasse 129, Cologne, 50933 Germany; Department of Health Management and Policy, The University of Michigan School of Public Health, 1420 Washington Heights, Ann Arbor, MI 48109-2029 USA; German Cancer Society, Kuno-Fischer-Straße 8, 14057 Berlin, Germany; University of Education Freiburg, Kunzenweg 21, 79117 Freiburg, Germany

**Keywords:** Health literate health care organization, Instrument development, Health literacy, Hospitals, Cancer care

## Abstract

**Background:**

While research on individual health literacy is steadily increasing, less attention has been paid to the context of care that may help to increase the patient’s ability to navigate health care or to compensate for their limited health literacy. In 2012, Brach et al. introduced the concept of health literate health care organizations (HLHOs) to describe the organizational context of care. This paper presents our effort in developing and validating an HLHO instrument.

**Method:**

Ten items were developed to represent the ten attributes of HLHO (HLHO-10) based on a literature review, an expert workshop, a focus group discussion, and qualitative interviews. The instrument was applied in a key informant survey in 51 German hospitals as part of a larger study on patient information and training needs (PIAT-study). Item properties were analyzed and a confirmatory factor analysis (CFA) was conducted to test the instrument’s unidimensionality. To investigate the instrument’s predictive validity, a multilevel analysis was performed that used the HLHO-10 score to predict the adequacy of information provided to 1,224 newly-diagnosed breast cancer patients treated at the sample hospitals.

**Results:**

Cronbach’s α of the resulting scale was 0.89. CFA verified the one-factor structure after allowing for the correlation for four pairs of error terms. In the multilevel model, HLHO-10 significantly predicted the adequacy of information as perceived by patients.

**Conclusion:**

The instrument has satisfactory reliability and validity. It provides a useful tool to assess the degree to which health care organizations help patients to navigate, understand, and use information and services. Further validation should include participant observation in health care organizations and a sample that is not limited to breast cancer care.

## Background

The issue of health literacy has gained increased attention ever since the U.S. Department of Education issued a report in 1993 that showed a significant portion of the country’s adult population may have too limited literacy skills to comprehend written information needed for dealing with daily activities including health care [1]. Early research on health literacy included the development of instruments to adequately assess individuals’ health literacy level [2,3], analyses of the effects of low health literacy on disease knowledge, health behaviors, and other health outcomes [4], and interventions to mitigate those adverse effects e.g. [4,5]. By and large, research suggests that improving individual health literacy may not only produce better health outcomes [6,7] but also decrease health care costs [8-10], thereby saving resources to improve health at a population level. Recently, efforts have shifted to develop short and group-specific instruments to assess health literacy [11-14] and to implement measures that help individuals with specific health conditions or from especially vulnerable groups e.g. [15-17].

In Europe, health literacy research has advanced rapidly through major projects such as the European Health Literacy Survey (HLS-EU) that involves eight EU member states [18] and as part of a research initiative by the German Federal Ministry of Health/National Cancer Plan [19]. The HLS-EU study revealed that, as in the U.S., a large proportion of the population does not have adequate health literacy and that variation exists between countries participating in the project. The HLS-EU project follows a broad definition of health literacy proposed by Sørensen and colleagues, which covers a wide range of aspects mentioned in earlier definitions. According to Sørensen et al.’s definition, health literacy is:

“linked to literacy and entails people’s knowledge, motivation and competences to access, understand, appraise, and apply health information in order to make judgments and take decisions in everyday life concerning healthcare, disease prevention and health promotion to maintain or improve quality of life during the life course [20: p.3].”

With the increase of research on individual health literacy, attention has also shifted to the specific context in which care is provided. The Healthy People 2010 health literacy action plan and the Institute of Medicine 2004 report [21,22] laid the foundation for the recent focus on care context. The U.S. National Action Plan to Improve Health Literacy [23] advanced the perspective that context and individual skills both matter in affecting the care for patients. Specifically, Baker [24], Nutbeam [25], Rudd [26-29] and colleagues pointed out that individual health literacy is conditional and contextual, because patients’ ability to understand medical information and navigate the care-seeking process is related to the demands that health delivery systems place on them and because the challenges that each patient experiences in the care process can only be understood within the organizational context of care. Alternatively, the specific organizational context where care is provided, such as a physician practice or hospital, may contribute to compensating for patients’ limited health literacy. Many health literacy-related interventions – such as using media other than written information to convey health information, designing plain language information or consent forms, and equipping premises with easy to understand signposts and directories – are applicable in any health care organization [30,31]. However, such interventions require additional resources and have not yet been implemented in all settings. Besides helping patients navigate the health care organization, these measures themselves might contribute to increasing patients’ individual health literacy.

To advance research on care context and health literacy, Brach et al. proposed the concept of health literate health care organizations (HLHOs) to characterize and assess how health care organizations perform in dealing with patients’ health literacy issues [32]. Referring to earlier work by the Institute of Medicine [22,33] and others e.g. [24,27], cf. 32 for an extensive list, they contended that health literacy research and interventions should take the demands of the health care system into account and endeavor to ‘tackle system-level factors’ [32: p.1]. They defined HLHOs as health care organizations that “make it easier for people navigate, understand, and use information and services to take care of their health” [32: p.2]. Specifically, they proposed that HLHOs displayed the ten attributes listed in below.

Ten attributes of a health literate health care organization [32: p.3]

1. Has leadership that makes health literacy integral to its mission, structure, and operations.
2. Integrates health literacy into planning, evaluation measures, patient safety, and quality improvement.
3. Prepares the workforce to be health literate and monitors progress.
4. Includes populations served in the design, implementation, and evaluation of health information and services.
5. Meets the needs of populations with a range of health literacy skills while avoiding stigmatization.
6. Uses health literacy strategies in interpersonal communications and confirms understanding at all points of contact.
7. Provides easy access to health information and services and navigation assistance.
8. Designs and distributes print, audiovisual, and social media content that is easy to understand and act on.
9. Addresses health literacy in high-risk situations, including care transitions and communications about medicines.
10. Communicates clearly what health plans cover and what individuals will have to pay for services.

To date, there is no instrument for measuring the degree of implementation of these attributes in health care organizations. An adequate assessment of the attributes would need to reflect reliably the extent of their implementation in health care organizations and to demonstrate that they indeed are linked to improved outcomes as perceived by patients.

This paper presents the development of an instrument for assessing the degree to which the 10 HLHO attributes are implemented in health care organizations. The instrument was psychometrically validated using data from surveys of key informants and patients in German breast care center hospitals. To satisfy as a psychometrically valid scale, the 10 items we developed should reliably and consistently measure a common, latent variable – i.e., HLHO (the property of unidimensionality). We assessed reliability (internal consistency) and unidimensionality of the HLHO-10 measure and tested its criterion and predictive validity.

## Method

### Data collection and samples

Data were collected as part of a larger study (‘Strengthening patient competence: Breast cancer **p**atients’ **i**nformation **a**nd **t**raining needs’ (“PIAT”)). The study was conducted in a sample of German breast cancer center hospitals. We included hospitals that were certified according to the criteria of the German Cancer Society and the German Society for Senology [34] as of May 31, 2012. We excluded hospitals that took part in a mandatory patient survey conducted in one federal state (North Rhine-Westphalia) [35] to avoid surveying patients twice. A total of 247 breast cancer center hospitals met these inclusion criteria. From these hospitals, we randomly selected 98 to participate in the study. Fifty-six (57%)^a^ of the 98 hospitals consented to voluntarily participate in the study. The main reason of refusal was that the hospital already participated in another patient survey.

We used self-administered questionnaires to collect data from hospital directors (or their proxies) – the key informant survey – and patients – the patient survey. The study protocol was approved by the Ethics Committee of the Medical Faculty of the University of Cologne.

#### Hospital key informant survey

The key informant survey was conducted between June 26 and August 31, 2013. We mailed the questionnaire to a representative (the director of the breast center or a designated proxy) of each of the 56 consenting hospitals and asked them to fill out and return the questionnaire. The questionnaire contained the items we designed to assess HLHO (to be described in detail in Section HLHO items) as well as questions regarding other structural and process characteristics of the hospital (e.g., size, teaching status, ownership). The survey was designed according to the Dillman’s method – that is, we made three mail contact attempts [36], plus a final telephone reminder. Fifty-one of the consenting 56 hospitals returned the questionnaire (91%). These 51 hospitals make up the sample for analysis at the hospital level.

#### Patient survey

The patient survey was conducted in the 56 consenting hospitals between February 1 and August 31, 2013. Patients were included if they: (1) had undergone inpatient surgery for newly diagnosed breast cancer between February 1 and August 31, 2013; (2) had at least one malignancy; and (3) had at least one postoperative histological evaluation. Shortly after the surgery and at the end of their hospital stay, eligible patients were asked to give written consent to participate in the survey. Once they had consented, the questionnaire was handed to them to be filled out shortly before discharge, i.e. at the end of their hospital stay. In addition to the survey responses given by patients, participating hospitals provided information on each surveyed patient’s disease and treatment characteristics (e.g., cancer stage, type of surgery). Patients were also followed with additional surveys at 10 and 40 weeks after discharge. For the purpose of this study, we used only data obtained from the first wave of patient survey – the survey right before the hospital discharge.

Of the 1,846 patients meeting the eligibility criteria, 1,543 consented to the study (83.6%). Of these, 1,359 returned the questionnaire before the discharge (88.1%). Five responses were deemed unusable because they missed the hospital identifier and could not be matched to the hospital data. Of the remaining responses, 1,224 were treated in one of the 51 hospitals responding to the key informant survey and these patients made up the patient sample for the study. Our analysis showed that patients in the study sample did not differ from patients treated in the five hospitals that did not return the key informant survey with regard to cancer stage, age and education.

### Measures

#### HLHO items

We employed a mixture of methods to develop items for assessing HLHO. First, we drafted a pool of provisional items based on the Brach et al. paper [32] and earlier research e.g. [21-29], a thorough review of the literature on health literacy and context as mentioned in the introduction, and a focus group in October 2012 with six representatives from different breast care center hospitals that discussed the role of hospitals in providing patients with adequate information and addressing poor individual literacy. Second, we held a workshop with employees of breast care center hospitals in January 2013 to discuss and select items that best reflected hospitals’ implementation of the 10 HLHO attributes defined in Brach et al. [32]. The workshop participants (N = 15) included quality managers (1), doctors (2), registered/specialist nurses (9), center coordinators (2), and self-help representatives (1). A consensus was reached that the final set of items should be parsimonious, easily understandable to respondents, and pertinent to the practices in German breast cancer center hospitals. The discussion at the workshop resulted in a draft of 10 items, with one item measuring each of the 10 HLHO attributes. Third, the 10 items were then reviewed by researchers from different disciplines (nursing, sociology, psychology, health economics), many of whom trained in developing survey questions to improve the wording and to ensure face validity.

Each of the final set of items (Table 1) was answered on a seven-point scale, ranging from ‘not at all’ to ‘to a very large extent’. This scale format was chosen to reflect the continuous character of each of the 10 attributes and to avoid the agree/disagree format [37]. Note that the order of items in Table 1 differs from the order of HLHO attributes appeared in Brach et al. [32] (see list above). The third attribute (‘workforce’) is represented by the tenth item in Table 1. Participants in the January 2013 workshop were perplexed by the workforce question and later discussion suggested that the issue should be addressed later in relation to other HLHO attributes. Moreover, a short introduction was added, which included a brief (perhaps oversimplified) definition of health literacy to familiarize respondents with the concept and the role of hospitals in promoting patient health literacy.

**Table 1:** **HLHO**-**10 items**

**Health literacy as a topic at your location**								
Patients have varying levels of health literacy. Health literacy is the ability to find, understand and put health information into practice. The following statements relate to measures at your hospital, which consider and promote the health literacy of your patients. Please think about your hospital in answering the questions. Please assess your hospital in accordance to each question on a scale from 1 ‘absolutely not’ to 7 ‘to a very large extent’.								
To what extent								
**Item** (attribute no., aspect)	**M**	**SD**	**Md**	**S**	**Min**	**Max**	**r**_**it**_	**P**_**i**_
…is the management at your hospital explicitly dedicated to the subject of health literacy (e.g. mission statement, human resources planning)? (1, leadership)	5.00	1.50	5.0	−0.894	1	7	0.768	0.67
…is the topic of health literacy considered in quality management measures at your hospital? (2, integration)	4.96	1.73	5.0	−0.779	1	7	0.751	0.66
…is health information at your hospital developed by involving patients? (4, inclusion of the served)	3.70	1.72	4.0	−0.040	1	7	0.622	0.45
…is individualized health information used at your hospital (e.g. different languages, print sizes, braille)? (5, health literacy skills range)	3.57	1.65	4.0	0.179	1	7	0.634	0.43
…are there communication standards at your hospital which ensure that patients truly understand the necessary information (e.g. translators, allowing pauses for reflection, calling for further queries)? (6, communication standards)	5.35	1.37	6.0	−0.970	1	7	0.710	0.73
…are efforts made to ensure that patients can find their way at your hospital without any problems (e.g. direction signs, information staff)? (7, provide access)	5.75	1.31	6.0	−1.620	1	7	0.533	0.79
…is information made available to different patients via different media at your hospital (e.g. three-dimensional models, DVDs, picture stories)? (8, media variety)	3.98	1.85	4.0	−0.108	1	7	0.511	0.50
…is it ensured that the patients have truly understood everything, particularly in critical situations (e.g. medication, surgical consent), at your hospital? (9, high-risk)	6.04	0.95	6.0	−0.683	1	7	0.439	0.84
…do you communicate openly and comprehensibly at your hospital to your patients in advance about the costs which they themselves have to pay for treatment (e.g. out-of-pocket payments)? (10, costs)	5.78	1.31	6.0	−0.895	1	7	0.462	0.80
…are employees at your hospital trained on the topic of health literacy? (3, workforce)	4.38	1.65	4.5	−0.337	1	7	0.833	0.56

*M* = Mean, *SD* = Standard deviation, *Md* = Median, *S* = Skewness, *Min* = Minimum, *Max* = Maximum, r_it_ = Discrimination (corrected item-total-correlation), P_i_ = Difficulty; N = 51.

#### Other hospital characteristics

In order to identify patterns of HLHO implementation and to adjust for relevant hospital differences, the ‘conventional set’ [38] of hospital structure characteristics was assessed in the hospital questionnaire, including teaching status (non-teaching vs. teaching), ownership status (public, charitable, for-profit), and patient volume (annual number of surgeries performed on newly diagnosed breast cancer patients).

#### Patient characteristics

To assess the predictive validity, we used information from the patient survey to construct a variable regarding the adequacy of information provided in the breast care hospital. The variable was based on eight items asking patients their perceived adequacy of information that they received from the hospital regarding: (1) breast cancer self-help groups; (2) psychological support programs; (3) rehabilitation possibilities; (4) “patient guideline”, a brochure by the German Cancer Society and the German Cancer Aid; (5) obtaining a second opinion from another doctor; (6) dealing with side effects of treatment; (7) possible critical incidents that may occur at home; and (8) activities that should be avoided during treatment.

For each item, there were six possible answers (‘I received too little information’ , ‘the information was exactly right for me’ , ‘I received too much information’ , ‘I was over-challenged with the information’ , ‘I wasn’t offered information’ , and ‘I didn’t want any information’). ‘The information was exactly right for me’ was coded 1; ‘I didn’t want any information’ was coded missing; and all other answers were coded 0. Exploratory factor analysis of the eight items (principal component extraction method with varimax rotation) suggested two latent factors, whose initial eigenvalues were 3.4 and 1.2, respectively. Because the second factor had a small eigenvalue (barely larger than 1), we decided to use the average of responses to all eight items to present the perceived adequacy of information provided by the hospital. The composite variable was constructed for patients with a least five valid (i.e. non-missing) answers. The Cronbach’s alpha of the variable was 0.81, suggesting satisfactory internal reliability.

Patient attributes may be related to different information demands. Our analysis accounted for patient age (categorized into younger than 40 years, 41 to 50, 51 to 60, 61 to 70, over 71), type of surgery (mastectomy vs. breast conserving treatment), UICC cancer stage (stages 0-I vs. stages II-IV) [39], and health literacy. Health literacy was assessed using the three ‘best performing’ items provided by Chew et al. [12,13], which have been widely used in surveys [40,41]. We used the mean value of the items for patients with at least two valid answers to represent health literacy (Cronbach’s α = .75).

### Validity and reliability assessment

We imputed the missing values on the HLHO items using the expectation-maximization (EM) algorithm in the software NORM [42,43]. The EM algorithm estimates missing data using an iterative maximum-likelihood estimation procedure [44].

We employed classical measurement theory to validate the 10-item instrument. In classical measurement theory, the two key psychometric properties of an instrument are its reliability, defined as the extent to which the instrument produces consistent results, and validity, the degree to which the instrument measures what it purports to measure. In assessing the psychometric properties of the instrument, we assumed the 10 items contributed to a total measure of the concept, Health Literate Healthcare Organization. On the basis of this assumption, the following four steps were taken to assess the reliability and validity of the instrument. First, we performed item analysis to examine the extent to which each item was correlated with the score of the total instrument. Each item’s relationship with the total score was assessed using corrected item-total correlation. In addition, we calculated the Cronbach’s alpha to examine the internal consistency of the items, or the degree to which hospital key informants answered consistently on the 10 items.

Second, we performed exploratory factor analysis to confirm the existence of a dominant latent factor and confirmatory factor analysis to confirm the unidimensionality of the ten items. To assess the factor structure of the item set, a principal components analysis with varimax rotation was performed. The global fit of a one-factor model in confirmatory factor analysis was assessed using the following measures and criteria: a non-significant chi square value (p > 0.05), a root mean square error of approximation (RMSEA) value of <0.08, and comparative fit index (CFI) and Tucker-Lewis Index (TLI) values of ≥0.90 [45].

Third, we performed several bivariate tests to examine criterion validity and to identify patterns of HLHO implementation among German breast cancer center hospitals. We calculated Spearman’s rho to examine the correlation between HLHO-10 and hospital volume. After testing of normality (Shapiro-Wilk test) and homogeneity of variance (Levene statistic), we conducted t-test and ANOVA to examine differences in HLHO-10 by teaching and ownership status.

Finally, to assess predictive validity, we tested in a hierarchical linear model of whether hospitals’ HLHO-10 score was positively correlated with the perceived adequacy of information provided to patients [46]. Both the HLHO-10 score and the adequacy of information variable were transformed into z-scores to facilitate interpretation [47]. In performing the hierarchical linear analysis, we first fit the two-level model without predictors (null model) to calculate an intraclass correlation coefficient (ICC). The ICC of the null model represents the proportion of variance in the dependent variable attributable to the hospital level. Following this, both patient characteristics and HLHO-10 (grand-mean centered) were added to the model to test the association between HLHO-10 and the extent to which patients consider the information provided as adequate. To account for hospital-level differences, hospital volume, teaching status, and ownership status were added to the model in a final step. Variables representing missing values on categorical variables were included in the models but omitted in the results presented below. Observations with missing values on continuous variables (i.e., health literacy, adequacy of information provided) were excluded from the analysis. IBM SPSS Statistics 22.0 was used for descriptive analysis, IBM SPSS AMOS 22.0 for the structural equation modeling, and HLM 7 for multilevel analysis.

## Results

Characteristics of the patients included in the analysis are presented in Table 2. Approximately half of the patients in the sample had stages 0/I or II-IV cancer and 78% received breast conservation treatment. Patients’ mean age was 59 with most patients in the 51 to 60 year old group.

**Table 2:** **Patient characteristics** (**n** = **1**,**224**)

	**Valid percent** **(n)**
Stage	
Stage 0-I	47.5 (504)
Stage II-IV	52.5 (558)
Missing	(162)
Type of surgery	
Mastectomy	22.0 (254)
Breast conserving treatment	78.0 (899)
Missing	(71)
Age	
20-40	4.6 (55)
41-50	23.3 (281)
51-60	28.1 (339)
61-70	26.3 (317)
≥70	17.8 (215)
Missing	(17)
Health literacy (mean, SD, median)	(3.58, 0.85, 3.67)

The items of the HLHO-10 instrument, their means, medians, skewness, minimum and maximum, corrected item-total correlation, and difficulty are displayed in Table 1. The ten HLHO items showed acceptable to good item-total correlations of between 0.44 and 0.83 and difficulties between 0.43 and 0.84. The Cronbach’s alpha of HLHO-10 was 0.89. Together, these findings suggest satisfactory internal reliability.

**Figure 1:**
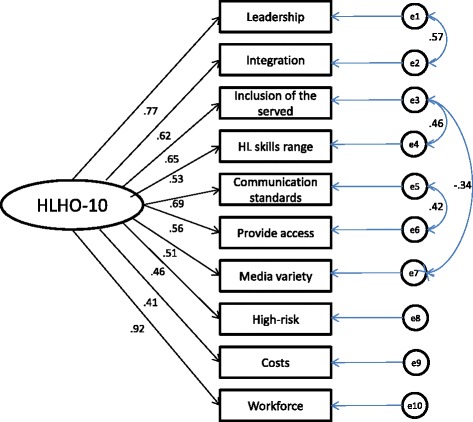
**Confirmatory factor analysis.**

Exploratory factor analysis with varimax rotation revealed a two-factor structure. However, the first factor explained 51% of total variance, which was 4.25 times that of the variance explained by the second factor (eigenvalue = 1.2; 12% of total variance), suggesting the existence of a dominant latent factor. Confirmatory factor analysis of the one-factor model (Figure 1) showed an acceptable fit after allowing for the correlation of four pairs of error terms (‘leadership’/’integration’; ‘inclusion of the served’/’health literacy skills range’; ‘communication standards’/’provide access’; ‘inclusion of the served’/’media variety’), resulting in a model with χ2/df [39.477/31] = 1.27, RMSEA: .073; TLI: .952; CFI: .967 (Table 3). Taken together, these results verify the property of unidimensionality – that is, the 10 items contribute to a total measure of HLHO.

**Table 3:** **Measures of Global Fit Confirmatory Factor Analysis**

	**χ 2**	**d.f.**	**p**	**Х** ^**2**^ **/d.f.**	**TLI**	**CFI**	**RMSEA**
Thresholds for acceptable fit			> 0.05	<3	≥0,90	≥0,90	<0,08
Original model	80.917	35	<0.001	2.312	0.768	0.820	0.159
Modified model	39.477	31	0.141	1.273	0.952	0.967	0.073

*CFI*, comparative fit index; *RMSEA*, root mean square error of approximation; *TLI*, Tucker-Lewis Index.

The Shapiro-Wilk test statistic was non-significant for the HLHO-10 score, suggesting normality and homogeneity of variance (p-value = 0.638; df = 51). No difference in HLHO-10 was found by teaching (t-test, P = 0.968) and ownership (ANOVA, P = 0.512) status; neither was there a significant association between HLHO-10 and patient volume (Spearman’s r, P = 0.361) (Table 4).

**Table 4:** **Hospital characteristics and bivariate associations with HLHO**-**10**

**HLHO**-**10**			
	**n**		**P-value**
All hospitals		4.86	
Teaching hospital			0.968
Yes (mean)	44	4.86	
No (mean)	7	4.84	
Ownership status			0.512
Public (mean)	28	4.81	
Charitable (mean)	16	5.08	
For-profit (mean)	7	4.53	
Patient volume (Spearman’s r)		−0.131	0.361

Note: P-values based on t-test (teaching status), ANOVA (ownership status) and Spearman’s r (patient volume).

The ICC of the null model was 0.04. It decreased after adding HLHO-10 and patient control variables (Table 5). HLHO-10 was significantly and positively associated with the dependent variable (p < 0.05), suggesting that an increase in the implementation of the HLHO attributes was related to a better perception of information adequacy among breast cancer patients treated at the hospital. Among the patient characteristics, only individual health literacy was significantly associated with perceived adequacy of the information provided. These associations persisted after the inclusion of control variables at the hospital level, none of which was statistically significant at p < 0.05. In additional analyses patient characteristics (including education, native language, partnership status, and type of health plan) were not significantly associated with the dependent variable.

**Table 5:** **Results of the hierarchical linear regression models on perceived adequacy of information**

	**Model 1**	**Model 2**
Intercept	0.02 (0.738)	0.19 (0.094)
**Patient characteristics**		
Stages II-IV (vs. 0-I)	−0.00 (0.991)	−0.00 (0.941)
Mastectomy (vs. bct)	0.02 (0.776)	0.02 (0.748)
Age groups (ref. 61 to 70)		
≤40	−0.03 (0.871)	−0.02 (0.891)
41 to 50	−0.01 (0.912)	−0.01 (0.903)
51 to 60	0.02 (0.827)	0.01 (0.874)
≥71	−0.08 (0.400)	−0.09 (0.355)
Health literacy	0.15 (<0.001)***	0.15 (<0.001)***
**Hospital characteristics**		
Teaching		−0.21 (0.069)
Patient volume		−0.00 (0.084)
Ownership (ref. public)		
Charitable		0.01 (0.942)
For-profit		0.13 (0.223)
HLHO-10	0.08 (0.032)*	0.09 (0.031)*
*Variance components for random effects*:		
Between-hospital variance (τ_00_): SD	.04; .19***	.04; .19**
Degrees of freedom	49	45
Chi-square	91.66	80.14
ICC (FUM: .040)	.038	.037

Fixed effects with robust standard errors; b (P-value); n = 1,154 patients; N = 51 hospitals; *p < .05; **p < .01; ***p < .001.

## Discussion

Overall, our analysis showed that the ten-item instrument we developed based on the Brach et al. [32] framework had acceptable to good psychometric properties. Importantly, the multilevel model found a significant association between HLHO-10 and patients’ perceived adequacy of information provided by hospitals, adjusted for patient and hospital characteristics. In addition to verifying the instrument’s predictive validity, the result is indicative of the usefulness of the HLHO concept in explaining patient experience.

There are two basic ways to use HLHO-10. First, it can be used in research as a measurement tool to assess the extent to which hospitals, and possibly other healthcare organizations, are a health-literate healthcare organization and their ability to deal with patients’ health literacy constraints. Second, healthcare organizations can use HLHO-10 as a self-assessment tool to identify areas that need improvement and to devise plans to improve their ability to address patients’ health literacy issues. Besides assessing patient perception of care, future research may try to assess the impact of health literacy in health organizations on objective changes in care delivery as well as on health outcomes.

A few caveats of the study need to be discussed. First, the Brach et al. [32] paper describes the ten HLHO attributes in a lot more detail than they are operationalized here. Our decision to “simplify” the items used in the instrument was based on the considerations of designing items that were relevant and understandable to hospital key informants and selecting items that were actionable. Second, the items were developed within the setting of the German health care system and, more specifically, within the setting of breast cancer care. Breast cancer care in Germany is highly standardized, not only with regard to guideline adherence and staffing, but also with regard to the emphasis on patient-centeredness, which is acknowledged and emphasized in the certification processes [34,35]. This may explain why we did not find significant differences in HLHO by hospitals’ patient volume, ownership status and teaching status. The fact that the items were developed in the specific context of the study also suggests that further work may be needed to test the utility of the instrument in other countries, other care domains, and in other types of healthcare organizations. Third, the assessment of HLHO was conducted using data from a survey of hospital key informants – directors of breast cancer hospitals or their designated proxies. We are unsure if the HLHO assessment results may be different from the viewpoints of other stakeholders. Further validation of the instrument would therefore be needed by using, for example, participant observations or researchers assessing the HLHO attributes independent of hospital representatives. Fourth, the study sample consisted of 51 breast cancer hospitals. The small sample size may be another explanation of the lack of significant association between HLHO and patient volume, hospital ownership, and teaching status. Further assessment of the instrument using larger and more diverse samples of healthcare organizations is warranted.

The differences in the extent to which the attributes are already implemented require the analysis of facilitators and barriers for change at an organizational level. A recent value-oriented investigation showed only marginal associations with culture change measures in U.S. nursing homes [48] and earlier attempts to track or systematically influence organizational change did not exactly result in step-by-step guidelines [49]. The conceptual development of HLHOs has so far resulted in a well-received discussion paper that launched a number of research initiatives. The ten attributes stated in the paper were meant to exemplify HLHOs and not to be exhaustive. When revising, adding, or prioritizing the attributes, it is preferable to emphasize the function of compensating for impaired individual health literacy – that is, to put effort into those who are in most need of health literacy-related support. The work we present in this paper represents one of few current attempts that relate individual health literacy issues to efforts at the organizational level.

## Conclusion

The HLHO-10 instrument provides a useful tool to assess the degree to which healthcare organizations focus on dealing with patients’ health literacy issues. Further validation should include alternative data collection methods (e.g., participant observation in healthcare organizations) and larger and more diverse samples.

## Endnote

^a^Breast care centers may consist of one to four hospitals, with the majority of breast centers consisting of one. Analyses reported here are done at the hospital level, not the breast care center level.
